# QL1209 (pertuzumab biosimilar) versus reference pertuzumab plus trastuzumab and docetaxel in neoadjuvant treatment for HER2-positive, ER/PR-negative, early or locally advanced breast cancer: A multicenter, randomized, double-blinded, parallel-controlled, phase III equivalence trial

**DOI:** 10.1038/s41416-024-02751-2

**Published:** 2024-06-21

**Authors:** Wenjia Zuo, Zhonghua Wang, Jun Qian, Xiaopeng Ma, Zhaofeng Niu, Jianghua Ou, Qinguo Mo, Jing Sun, Xinzheng Li, Qitang Wang, Yongzhong Yao, Guohua Yu, Hongsheng Li, Dedian Chen, Hao Zhang, Cuizhi Geng, Guangdong Qiao, Mengmeng Zhao, Baihui Zhang, Xiaoyan Kang, Jin Zhang, Zhimin Shao

**Affiliations:** 1 Department of Breast Surgery, Fudan University Shanghai Cancer Center, Shanghai, 200032 China; 2 Department of Surgical Oncology, The First Affiliated Hospital of Bengbu Medical College, Bengbu, 233099 China; 3 Department of General Surgery, The First Affiliated Hospital of USTC, Division of Life Science and Medicine, University of Science and Technology of China, Hefei, 230001 China; 4 Department of Breast Disease, Yuncheng Central Hospital, Yuncheng, 044099 China; 5 Department of Breast Surgery, Cancer Hospital Affiliated to Xinjiang Medical University, Urumqi, 830000 China; 6 Department of Breast Surgery, Guangxi Medical University Cancer Hospital, Nanning, 530021 China; 7 The Fifth Department of Internal Medicine, Anyang Tumor Hospital, Anyang, 455000 China; 8 Department of Breast Surgery, Shanxi Cancer Hospital, Xi’an, 710061 China; 9 Breast Medical Center, Qingdao Central Hospital, Qingdao, 266042 China; 10 Department of Breast Surgery, Department of General Surgery, Nanjing Drum Tower Hospital, the Affiliated Hospital of Nanjing University Medical School, Nanjing, 210008 China; 11 Department of Oncology, Weifang People’s Hospital, Weifang, 261071 China; 12 Department of Breast Surgery, Affiliated Cancer Hospital and Institute of Guangzhou Medical University, 511436 Guangzhou, China; 13 The Second Department of Breast surgery, Yunnan Cancer Hospital, Kunming, 650118 China; 14 Department of Breast Surgery, Nanyang Central Hospital, Nanyang, 473005 China; 15 Breast Center, The Fourth Hospital of Hebei Medical University, Shijiazhuang, 050011 China; 16 Department of Breast Surgery, Yantai Yuhuangding Hospital, Yantai, 264099 China; 17 Clinical Research Center, Qilu Pharmaceutical Co., Ltd, Jinan, 250105 China; 18 The Third Department of Breast Cancer, Tianjin Medical University Cancer Institute & Hospital, Tianjin, 300060 China

**Keywords:** Breast cancer, Cancer therapy

## Abstract

**Background:**

This randomized, parallel-controlled, double-blinded, phase III equivalence study evaluated the equivalence of a proposed pertuzumab biosimilar QL1209 to the pertuzumab (Perjeta®) each with trastuzumab and docetaxel in neoadjuvant treatment of early or locally advanced breast cancer patients with HER2-positive, ER/PR-negative.

**Methods:**

Eligible patients were randomly (1:1) assigned to receive 4 cycles of neoadjuvant QL1209 or pertuzumab each with trastuzumab and docetaxel, and adjuvant treatment. The primary endpoint was total pathologic complete response (tpCR), with equivalence margins of 0.76 to 1.32.

**Results:**

Among the 585 patients enrolled, 257 and 259 patients were assigned to the QL1209 and pertuzumab groups, respectively. The tpCR rates were comparable in the QL1209 (109/255, 42.75%; 90% CI 37.65 to 47.84) and pertuzumab (117/259, 45.17%; 90% CI 40.09 to 50.26) groups. The tpCR risk ratio was 0.95 (90% CI, 0.80 to 1.11), and the 90% CI fell within the predefined equivalence margin. The most common grade ≥3 treatment-related adverse event was decreased neutrophil count (10. 9% vs. 12.7%) in the QL1209 and pertuzumab groups.

**Conclusions:**

QL1209 demonstrated equivalent efficacy and comparable safety profile to the reference pertuzumab in neoadjuvant treatment of HER2-positive, ER/PR-negative, early, or locally advanced breast cancer.

**Trial registration:**

Chinadrugtrials.org CTR20201073; ClinicalTrials.gov NCT04629846.

## Introduction

Human epidermal growth factor receptor 2 (HER2) overexpression or amplification occurs in approximately 15% to 20% of patients with breast cancer, associated with poor prognoses and a median overall survival of 25 months [1, 2]. The addition of pertuzumab to trastuzumab, in combination with chemotherapy, known as dual anti-HER2 therapy, has significantly enhanced the clinical benefit of neoadjuvant treatment in patients with HER2-positive breast cancer, revolutionizing the landscape of neoadjuvant therapy [3, 4]. This substantial clinical benefit is attributed to the synergistic efficacy of pertuzumab and trastuzumab, inhibiting HER2-HER3 dimerization and downregulating intracellular pathways such as phosphatidylinositol 3-kinase (PI3K/Akt) [5, 6]. Dual HER2 inhibition with trastuzumab and pertuzumab plus docetaxel has received approval as the standard of care for HER2-positive breast cancer in neoadjuvant, adjuvant, and first-line treatment by the US Food and Drug Administration (FDA) and the National Medical Products Administration (NMPA) of China, based on results from the NeoSphere, APHINITY, and CLEOPATRA trials, respectively [3, 7–9]. Pertuzumab finds widespread application in various clinical scenarios for patients with HER2-positive breast cancer. Adding pertuzumab to trastuzumab plus chemotherapy achieves pronounced efficacy in early or locally advanced breast cancer patients with HER2 positive, especially in patients with HER2 positive and ER/PR-negative status [3, 10]. However, the combination of pertuzumab with neoadjuvant trastuzumab and chemotherapy has been identified as a cost-saving option for specific subgroups of patients with HER2-positive breast cancer [11, 12]. However, in 2016, pertuzumab was reported as unlikely to be cost-effective at a willingness to pay of $100,000 per quality-adjusted life-year (QALY) gained for HER2-positive metastatic breast cancer, even with a reduction of more than 71% in the prices of pertuzumab and trastuzumab [13, 14]. Notably, the rate of pertuzumab utilization in neoadjuvant and adjuvant settings was much lower than in the metastatic setting, as recommended by the National Institute for Health and Care Excellence (NICE) in England [15]. The high cost and limited accessibility of pertuzumab may contribute, in part, to the gap between evidence and practice.

In China, breast cancer ranks the highest incidence rate among women, with an estimated 345.8 thousand diagnosed cases in 2023, and the fifth leading cancer by mortality rate from 2005–2020 in China [16, 17]. There is a substantial need for pertuzumab in patients with HER2-positive breast in China. At the initiation of our study, pertuzumab had been approved to treat breast cancer along with trastuzumab without medical insurance coverage in China [18]. There existed a significant unmet need for patients with HER2-positive breast cancer to access pertuzumab treatment in China, necessitating the urgent development of a pertuzumab biosimilar to overcome this dilemma [19, 20].

QL1209, developed by Qilu Pharmaceutical Co., Ltd, Jinan, China, is a proposed biosimilar to the reference pertuzumab (Perjeta®) and the first pertuzumab biosimilar produced and submitted for approval in China. It possesses an identical amino acid sequence and biosimilarity to pertuzumab [21]. In the preclinical study, QL1209 demonstrated a safety profile and pharmacokinetic (PK) profile similar to pertuzumab (data not published). In a phase I clinical trial involving healthy male volunteers, QL1209 exhibited PK equivalence and similar safety and immunogenicity compared to pertuzumab [21]. This study further investigates the similarity of QL1209 to reference pertuzumab in terms of efficacy and safety in the neoadjuvant treatment of early or locally advanced HER2-positive, ER/PR-negative breast cancer.

## Methods

### Study design and participants

In this randomized, double-blind, parallel-controlled, multicenter, phase III equivalence clinical trial, patients were recruited from 52 medical centers in China. Eligible participants were aged 18 to 75 years, with histologically confirmed HER2-positive breast cancer at accredited local laboratories. The study included patients with early (T2-3, N0-1, M0) or locally advanced (T2-3, N2 or N3, M0; T4, any N, M0) stage breast cancer, based on the 8th edition of the American Joint Committee on Cancer (AJCC) staging system for breast cancer [22], and with confirmed HER2-positivity and ER/PR negative statuses. Other inclusion criteria included an Eastern Cooperative Oncology Group performance status of 0 to 1 and a baseline left ventricular ejection fraction (LVEF) ≥55%, measured by echocardiography (first choice) or multigated acquisition scan. The main exclusion criteria included stage IV or bilateral breast cancer, prior antineoplastic treatment or radiation therapy for any malignancy, and pregnancy or lactation. The full inclusion and exclusion criteria are detailed in the methods section in the Supplementary Material.

The study received ethical approval from independent ethics committees for each center and adhered to the Declaration of Helsinki, Good Clinical Practice guidelines, and all applicable regulatory requirements. The study protocol, including subsequent modifications, was approved by ethics committees, and written informed consent was obtained from all participants.

### Randomization and Masking

Following eligibility confirmation, patients were centrally and randomly (1:1) assigned to one of two treatments: QL1209 (Qilu Pharmaceutical, Jinan, China) combined with trastuzumab (Roche, Basel, Switzerland) and docetaxel (Qilu Pharmaceutical, Jinan, China) for the QL1209 group, or pertuzumab (Roche, Basel, Switzerland) plus trastuzumab (Roche, Basel, Switzerland) and docetaxel (Qilu Pharmaceutical, Jinan, China) for the pertuzumab group. The allocation schedule was generated using central stratified randomization, stratified by disease stage (early-stage vs. locally advanced-stage, per the 8th AJCC staging system). As the study was double blinded, patients, investigators, study site personnel, review committee, and the sponsor’s study team were masked to treatment allocation.

### Procedures

This study was initially designed to investigate the equivalence of QL1209 to reference pertuzumab in efficacy and safety according to the 1.1 and 2.0 version of protocol. As the study progressed, adjuvant treatment was added post-surgery in the updated version 3.0 protocol, in accordance with the Guidelines for clinical trials of biosimilars for pertuzumab injection, released on April 21, 2021, by the Center for Drug Evaluation NMPA in China [23].

During the neoadjuvant period, patients underwent treatment with QL1209 or pertuzumab, combined with trastuzumab and docetaxel for 4 cycles (every 3 weeks). QL1209 or reference pertuzumab received an 840 mg loading dose in cycle 1, followed by 420 mg in cycles 2-4. Trastuzumab was administered at a loading dose of 8 mg/kg in cycle 1, followed by 6 mg/kg in cycles 2–4. Docetaxel was administered at 75 mg/m² immediately after QL1209 or reference pertuzumab in cycles 1–4. Dose adjustments were permitted at the investigator’s discretion, with discontinuation of QL1209/pertuzumab recommended if reference trastuzumab was discontinued. Surgery was performed within 2 weeks after completing the 4-cycle neoadjuvant treatment.

In the subsequent neoadjuvant period, patients received FEC chemotherapy (fluorouracil 500-600 mg/m², epirubicin 90-120 mg/m², cyclophosphamide 500–600 mg/m²) in cycles 5-7, along with QL1209 and trastuzumab at the previous doses in cycles 8–20. Treatment continued until the completion of 20 cycles or until an event occurred, such as disease recurrence, loss to follow-up, death, withdrawal of informed consent, switch to alternative tumor treatment, or intolerable toxicity, whichever occurred first. The end-of-treatment or treatment-discontinuation visit was scheduled 28 days after the final administration of the study drug, marking the conclusion of the study.

### Assessments

Laboratory parameters, including hematology and serum chemistries, physical examination, 12-lead electrocardiogram, Eastern Cooperative Oncology Group Performance Status (ECOG PS), and vital signs were assessed at each cycle. Routine tumor response, evaluated through clinical breast examination, was conducted every 2 cycles during the initial 4 treatment cycles, followed by assessments every 4 cycles from cycles 8 to 20, in accordance with Response Evaluation Criteria in Solid Tumors version 1.1 (RECIST v.1.1). A preoperative assessment, encompassing a physical examination, mammogram, and ultrasound (if deemed necessary according to local practice), was performed. The independent review committee (IRC) and investigators (INV) evaluated surgical specimens. Pathologic response was determined by local histopathology assessment of surgical breast specimens and lymph node tissues post neoadjuvant therapy, according to the 8th edition AJCC ypTNM staging system.

Adverse events (AEs) were monitored until hospital discharge post-surgery or 28 days after the last adjuvant treatment. AE severity was coded using MedDRA v25.0 and graded according to the National Cancer Institute common terminology criteria version 4.03 (NCI-CTCAE v4.03). Cardiac function, including LVEF and left ventricular systolic dysfunction (LVSD), was assessed using echocardiograms or multiple-gated acquisition scans at specified intervals. Symptomatic LVSD was defined as a symptomatic decrease in LVEF or explicit/highly likely cardiac-related death, while asymptomatic significant reduction in LVEF was defined as a decrease of ≥10% from baseline to an LVEF of ≤50%. Blood samples for PK and immunogenicity analysis were collected before each QL1209 or reference pertuzumab infusion at cycles 1-5, 10, 15, and the end of treatment/discontinuation visits.

### Endpoints

The primary endpoint was the total pathologic complete response (tpCR) rate assessed by IRC, defined as the absence of invasive tumor cells in the breast and ipsilateral axillary lymph nodes upon microscopic examination following primary tumor excision (ypT0/is, ypN0). Secondary endpoints included tpCR rate assessed by INV, breast pathological complete response rate (bpCR) based on IRC and INV, objective response rate (ORR), event-free survival (EFS), disease-free survival (DFS), safety, PK, and immunogenicity. The bpCR rate was defined as the absence of invasive tumor cells in the breast upon microscopic examination following primary tumor excision (ypT0/is). ORR was the percentage of patients with the best overall response of complete response (CR) or partial response (PR) according to RECIST v.1.1. EFS was the time from randomization to the first occurrence of disease progression, recurrence, or death from any cause, while DFS was the time from surgery to disease recurrence or death from any cause.

PK was assessed by minimum serum concentration (C_trough_). Immunogenicity analysis involves detecting antidrug antibodies (ADAs) and neutralization antibodies (NAbs). Treatment-induced ADA positivity was defined as ADA-positive after treatment in patients who were ADA-negative at baseline or had ADA-positive titers at baseline with an increase in ADA concentration of ≥4-fold post-baseline.

### Statistical analysis

A sample size of 512 patients was planned to achieve 80% power in detecting equivalence at a predefined margin, with a significance level determined by two one-sided tests (α = 0.025), assuming a dropout rate of 10% and an estimated 50% of patients achieving a tpCR. The sample size was calculated using PASS software (NCSS, LLC, Kaysville, UT). The equivalence margin was determined through a meta-analysis of efficacy data in the ER/PR-negative subgroup from the NeoSphere and PEONY studies [3, 10]. The pooled relative risk (RR) of tpCR for pertuzumab plus trastuzumab and docetaxel versus trastuzumab plus docetaxel was 2.11 (70% confidence interval (CI), 1.74-2.57). The chosen equivalence margin of 0.76 to 1.32 aimed to preserve at least half of the efficacy observed in these studies [23].

Analyses of the primary endpoint (tpCR) and secondary endpoints (bpCR, ORR, EFS, and DFS) were conducted in the full analysis set (FAS). A 2-sided 90% Wald CI for the ratio of tpCR rate was calculated using logarithmic transformation without covariate adjustment. Subjects with concomitant events were categorized as non-responders, and those with missing assessment results were not imputed. Equivalence was affirmed if the CI entirely fell within the range of 0.76 to 1.32. Time-to-event endpoints (EFS and DFS) were estimated using the Kaplan-Meier method, with 95% CIs, and inter-group comparisons were performed via the stratified log-rank test. RR and associated 90% CIs were assessed using a stratified Cox proportional-hazards model.

To ensure the robustness and avoid or minimize potential bias, we analyzed the primary endpoint in per-protocol set (PPS; including patients in the FAS who had completed primary efficacy evaluations, except those who had a major protocol deviation or did not receive 4 doses of study drug at least), performed supplementary analyses of the primary endpoint in patients with centrally confirmed HER2-positive and ER/PR-negative, conducted subgroup analyses according to stratification factor, tipping-point analyses. More detailed statistical analyses can be seen in the methods section in the Supplementary Material. Statistical analyses were performed using SAS software (SAS Institute) version 9.4 or later.

## Results

### Patients

**Fig. 1:**
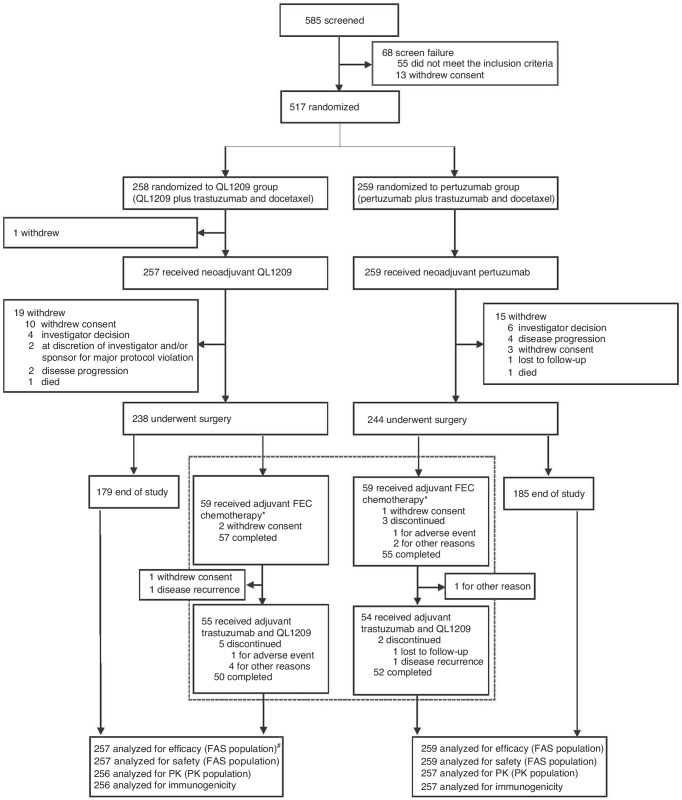
Trial profile. ^*^Patients who completed the neoadjuvant treatment and surgery received additional adjuvant treatment with 3 cycles of FEC chemotherapy and subsequent QL1209 and trastuzumab, according to the version 3.0 protocol. ^#^For tpCR and bpCR analysis, the number was 255, as 1 surgical specimen was lost, and 1 could n’t be delivered due to the COVID-19 pandemic. FEC fluorouracil, epirubicin, and cyclophosphamide, FAS full analysis set, PK pharmacokinetics.

Between November 2020 and May 2022, 585 patients underwent screening, with 517 subsequently randomized. One patient from this cohort did not receive QL1209 post-randomization. Therefore, a total of 516 patients were included in the full analysis set (FAS), with 257 assigned to the QL1209 group and 259 to the reference pertuzumab group (Fig. 1). Baseline demographic and disease characteristics were comparable between the treatment groups in the FAS population (Table 1). Overall, the median age of patients in QL1209 group and reference pertuzumab group were 53 years (range, 24 to 71), and 53 years (range, 25 to 74), respectively. The majority of patients had early-stage disease in both groups (62.6% vs. 63.7%). The median LVEF level at baseline in QL1209 group and reference pertuzumab group were 65% (range, 55-81) and 66% (range, 53-78.80), respectively.

**Table 1:** Patient demographic and baseline clinical characteristics (full analysis set).

	QL1209 (*N* = 257)	Reference pertuzumab (*N* = 259)
Age, years; median (range)	53 (24–71)	53 (25–74)
Gender, *n* (%)		
Male	0	0
Female	257 (100)	259 (100)
Race, *n* (%)		
Han	244 (94.9)	243 (93.8)
Other	13 (5.1)	16 (6.2)
BMI, kg/m^2^; median (range)	24.22 (17.6–33.6)	24.33 (17.0–37.5)
Breast cancer type, *n* (%)		
Early	161 (62.6)	165 (63.7)
Locally advanced	96 (37.4)	94 (36.3)
Clinical TNM stage		
II	134 (52.1)	140 (54.)
III	111 (43.2)	115 (44.4)
Other	12 (4.7)	4 (1.5)
Lymph node status		
Yes	193 (75.1)	193 (74.5)
No	64 (24.9)	66 (25.5)
Previous anti-tumor therapies, *n* (%)	1 (0.4)	0
LVEF, %; median (range)	65 (55-81)	66 (53-78.8)

*BMI* Body Mass Index, *LVEF* left ventricular ejection fraction.

Overall, 482 out of 516 patients (93.4%) underwent surgery as planned, with 238 (92.6%) in the QL1209 group and 244 (94.2%) in the reference pertuzumab group. According to the revised 3.0 version of the protocol, 129 patients in both groups (25.0%; 64 in the QL1209 group and 65 in the reference pertuzumab group) were enrolled to receive additional adjuvant treatment with FEC chemotherapy and subsequent QL1209 and trastuzumab. Among them, 118 patients received adjuvant FEC chemotherapy, with 112 (94.9%) completing the treatment (57 in the QL1209 group and 55 in the reference pertuzumab group). Furtherly, 109 out of 112 (97.3%) patients received adjuvant trastuzumab and QL1209 treatment, with 102 out of 109 (93.6%) completing the regimen-50 in the QL1209 group and 52 in the reference pertuzumab group. The patient’s disposition is illustrated in Fig. 1.

### Efficacy

Efficacy analysis was conducted in the FAS population. The proportions of patients achieving tpCR by IRC were comparable between the QL1209 and reference pertuzumab groups at 42.75% (109/255; 90% CI, 37.65 to 47.84) and 45.17% (117/259; 90% CI, 40.09 to 50.26), respectively. The RR of tpCR was 0.95 (90% CI, 0.80 to 1.11), with the CI entirely within the predefined equivalence margins of 0.76 to 1.32 (Table 2). Tipping-point analyses (Fig. S1), PPS analysis (Table S1), and supplementary analyses reaffirmed the equivalence between the two groups (Table S1). Additionally, subgroup analyses based on disease stage showed similar tpCR proportions (Table 2, Table S1).

**Table 2:** Efficacy results in full analysis set.

	QL1209 *n/N* (%, 90%)	Reference pertuzumab *n/N* (%, 90%)	Relative risk
Assessed by IRC			
tpCR	109/255 (42.75; 37.65 to 47.84)	117/259 (45.17; 40.09 to 50.26)	0.95 (0.80 to 1.11)
tpCR according to clinical stage			
Early stage	69/159 (43.40; 36.93 to 49.86)	80/165 (48.48; 42.09 to 54.88)	
Locally advanced	40/96 (41.67; 33.39 to 49.94)	37/94 (39.36; 31.07 to 47.65)	
bpCR	128/256 (50.00; 44.86 to 55.14)	134/259 (51.74; 46.63 to 56.84)	
Assessed by INV			
tpCR	109/257 (42.41; 37.34 to 47.48)	120/259 (46.33; 41.24 to 51.43)	0.92 (0.78 to 1.08)
bpCR	125/257 (48.64; 43.51 to 53.77)	133/259 (51.35; 46.24 to 56.46)	
ORR	212/257 (82.49; 78.59 to 86.39)	212/259 (81.85; 77.91 to 85.79)	

Data are *n* (%; 90% CI), *IRC* independent review committee, *INV* investigator, *tpCR* total pathological complete response, *bpCR* breast pathological complete response, *ORR* objective response rate.

For the secondary efficacy endpoints, the RR of tpCR per INV was 0.92 (90% CI, 0.78 to 1.08). Both the QL1209 and reference pertuzumab groups showed similar IRC-assessed bpCR rates at 50.00% (128/256; 90% CI, 44.86 to 55.14) and 51.74% (134/259; 90% CI, 46.63 to 56.84), respectively (Table 2). The ORR was comparable between the two groups, with 82.49% (90% CI, 78.59 to 86.39) in the QL1209 group and 81.85% (90% CI, 77.91 to 85.79) in the reference pertuzumab group. Furthermore, rates of radiographic complete response (CR) and partial response (PR) were similar between the two groups at 5.06% (13/257) vs. 5.02% (13/259) for CR and 77.43% (199/257) vs. 76.83% (199/259) for PR.

At the end of the study (October 2023), based on FAS, events for EFS occurred in 2.33% (6/257) of patients in the QL1209 group and 2.70% (7/259) in the reference pertuzumab group, with HR 0.86 (90% CI, 0.35 to 2.16). The analysis of DFS was conducted on patients who completed the surgery without residual lesions (QL1209 group, *n* = 60; reference pertuzumab group, *n* = 63) according to the revised 3.0 version of the protocol. There was one DFS event in each group. In addition, Median EFS or DFS was not reached in either group. Stratification demonstrated no differences in EFS or DFS Kaplan-Meier curves between patients with early-stage and locally advanced breast cancers in either the QL1209 or reference pertuzumab groups (Fig. S2).

### Safety

The safety analysis population included all patients in the FAS population, with 257 and 259 patients in each group. The safety profile reveals similar tolerability between the two groups (Table 3). The incidence of treatment-emergent adverse events (TEAEs) was comparable, with rates of 95.3% (245/257) in the QL1209 group and 96.1% (249/259) in the reference pertuzumab group. In the QL1209 group, the most frequent TEAEs were alopecia (106/257, 41.2%), decreased white blood cell count (96/257, 37.4%), diarrhea (91/257, 35.4%), decreased neutrophil count (90/257, 35.0%), anemia (82/257, 31.9%), and nausea (81/257, 31.5%). The profiles of treatment-related adverse events (TRAEs) were also similar between the QL1209 and pertuzumab groups. TRAEs of any grade were reported in 235 (91.4%) and 239 (92.3%) participants, with grade ≥3 TRAEs observed in 70 (27.2%) and 73 (28.2%) participants in the QL1209 and reference pertuzumab groups, respectively; Decreased neutrophil count (10.9% vs. 12.7%) and decreased white blood cell count (5.1% vs. 6.9%) were the most frequently (>5% of patients) grade ≥3 TRAEs reported event in QL1209 group and reference pertuzumab group. Treatment-related serious adverse events (TRSAEs) were reported in 5.1% of patients in the QL1209 group and 4.6% in the reference pertuzumab group. Elevated alanine aminotransferase was the most frequent TRSAE, with a similar incidence (1.2% vs. 1.5%) in both groups. The incidences of TRAEs leading to discontinuation were similar between the two groups, with 0.4% (1/257) in the QL1209 group and 0.8% (2/259) in the reference pertuzumab group. (Table 3) During the study, two deaths occurred, including one case of upper gastrointestinal bleeding (possibly related to dexamethasone, unrelated with QL1209, trastuzumab, or docetaxel according to the sponsor) in the QL1209 group and one case of suicidal behavior (impossibly related with the study drugs) in the reference pertuzumab group. One (1.56%) patient in the QL1209 group experienced symptomatic LVSD (grade 1) at the follow-up of the final Cycle 20, which was mild and needed no symptomatic treatment. One (1.56%) patient in the reference pertuzumab group experienced an asymptomatic significant decrease in LVEF (grade 1) at the follow-up of Cycle 16, and discontinued treatment of pertuzumab and trastuzumab thereafter. Infusion-related reactions were reported in 25 (9.7%) of 257 patients in the QL1209 group vs. 15 (5.8%) of 259 in the reference pertuzumab group.

**Table 3:** Summary of adverse events (safety set population).

	QL1209 (*n* = 257)	Reference pertuzumab (*n* = 259)
TEAEs, *n* (%)		
Any grade	245 (95.3)	249 (96.1)
Grade ≥3	94 (36.6)	97 (37.5)
Serious TEAEs	23 (8.9)	25 (9.7)
TRAEs, *n* (%)		
Any grade	235 (91.4)	239 (92.3)
Grade ≥3	70 (27.2)	73 (28.2)
TRSAEs	13 (5.1)	12 (4.6)
TRAEs leading to discontinuation	1 (0.4)	2 (0.8)
Infusion-related reaction	25 (9.7)	15 (5.8)
Death	1 (0.4)	1 (0.4)
TEAEs occurred in ≥10% patients in either group		
Alopecia	106 (41.2)	107 (41.3)
Decreased white blood cell	96 (37.4)	86 (33.2)
Diarrhea	91 (35.4)	89 (34.4)
Decreased neutrophil count	90 (35.0)	82 (31.7)
Anemia	82 (31.9)	93 (35.9)
Nausea	81 (31.5)	72 (27.8)
ALT increased	76 (29.6)	68 (26.3)
Infections	70 (27.2)	59 (22.8)
AST increased	57 (22.2)	49 (18.9)
Vomiting	53 (20.6)	48 (18.5)
Fatigue	52 (20.2)	55 (21.2)
Fever	34 (13.2)	29 (11.2)
Anorexia	34 (13.2)	41 (15.8)
Cough	28 (10.9)	23 (8.9)
Pain	26 (10.1)	18 (6.9)
Hypertriglyceridemia	23 (8.9)	29 (11.2)

*TEAE* treatment-emergent adverse event, *TRAE*, treatment-related adverse event, *TRSAEs*, treatment-related serious adverse events, *ALT* elevated alanine aminotransferase, *AST* elevated aspartate aminotransferase.

### PK

PK analyses involved 256 and 257 patients in the QL1209 and reference pertuzumab groups, respectively. No noticeable differences were observed in PK endpoints between the two groups throughout the neoadjuvant period (Fig. S3). Additionally, C_trough_ remained stable and similar between the two groups during cycles 2–4 (Table S2).

### Immunogenicity

A total of 513 patients were included in the immunogenicity analysis, with 256 in the QL1209 group and 257 in the reference pertuzumab group. During neoadjuvant and adjuvant chemotherapy periods, the incidence of treatment-induced ADA positivity and total ADA positivity (whether treatment-related or not) was similar between the two groups during the entire treatment period. (Table S3) Sensitivity analysis of ADA/NAb in PK, tpCR, and safety supported the equivalence of QL1209 to reference pertuzumab. (Table S4–S6).

## Discussion

The outcomes of this randomized, multicenter, double-blinded phase III trial robustly establish the therapeutic equivalence between QL1209 and reference pertuzumab as neoadjuvant treatments for patients with HER2-positive, ER/PR-negative early-stage or locally advanced breast cancer. The trial successfully met its primary endpoint, demonstrating an equivalent proportion of patients achieving tpCR with QL1209 compared to reference pertuzumab in the neoadjuvant setting, as evidenced by the 90% confidence intervals of bpCR risk ratio entirely falling within prespecified equivalence margins. Additionally, QL1209 exhibited similar pharmacokinetic characteristics and immunogenicity to reference pertuzumab.

This study underscores the equivalence of QL1209 to pertuzumab based on compelling evidence, employing agreed-upon primary endpoints, defined equivalence margins, and an optimal study population. Firstly, recognizing pCR as an appropriate surrogate endpoint for accelerated drug approval in early-stage breast cancer [24], this trial aligned with prior studies like NeoSphere and PEONY, where pertuzumab added to trastuzumab with docetaxel significantly improved pCR rates [3, 10]. In this study, the primary efficacy assessment focused on tpCR, in accordance with FDA recommendations for accelerated approval in high-risk, early-stage breast cancer [25, 26]. Simultaneous evaluation of tpCR (breast and axilla) and bpCR provided a comprehensive perspective. Supplemental tipping-point analysis further emphasized the strength of equivalence in efficacy [24, 27–29]. Secondly, adopting an equivalence margin of 0.76–1.32, derived from a meta-analysis of efficacy data in the ER/PR-negative subgroup from NeoSphere and PEONY studies, demonstrated meticulous consideration of precision range and regulatory precedents in China [3, 10]. Thirdly, the inclusion of patients diagnosed with HER2-positive and ER/PR-negative breast cancer, a subgroup likely to benefit most from pertuzumab and trastuzumab treatment, facilitated a meaningful comparison of efficacy between the biosimilar and reference drugs [3, 10]. Additional supplementary analyses based on re-assessment of HER2-positive and ER/PR-negative status by a central laboratory confirmed the equivalence in patients with confirmed HER2 status in the local laboratory. Furthermore, the study quantified the impact of unmeasured confounders through tipping-point analyses and conducted quantitative bias analyses for missing data. The analyses of missing data revealed highly unlikely scenarios for tipping points, reinforcing the robustness of the results.

The safety profile of the QL1209 group closely resembled that of the reference pertuzumab, aligning with published safety results for both pertuzumab and QL1209 [3, 28]. Despite the substantial clinical benefits, concerns lingered regarding potential cardiac toxicity associated with the trastuzumab and pertuzumab combination. The TRYPHAENA study, focusing on patients with HER2-positive early breast cancer, demonstrated high pCR rates (61.60%) and favorable cardiac safety, countering worries about heart failure risk [30]. Given this context, cardiac safety was a key focus from the study’s initiation, particularly considering the association of trastuzumab plus pertuzumab and docetaxel with increased heart failure risk in metastatic breast cancer [31]. Notably, the results indicated no significant difference in the low incidence of cardiotoxicity between QL1209 and pertuzumab. Moreover, the incidence of symptomatic LVSD or asymptomatic decrease in LVEF associated with the study drugs was low in this study, which was much lower than the 3.50% reported in the meta-analysis of pertuzumab for HER2-positive breast cancer [32, 33]. This variance may be attributed to the enrollment of patients with low cardiac risk in our study. Additionally, during the adjuvant treatment period, QL1209 was administered in both groups, and there was no difference in immunogenicity among patients in the reference pertuzumab group. QL1209 demonstrated favorable interchangeability in terms of safety while determining interchangeability in efficacy would necessitate a longer follow-up for EFS and DFS [19].

Based on these results, the announcement of the equivalence of QL1209 to reference pertuzumab in terms of both efficacy and safety is compelling. QL1209 exhibited a high pCR rate for early-stage or locally advanced HER2-positive, ER/HR-negative breast cancer. The probability of achieving pCR was a crucial parameter affecting the cost-consequence analysis of adding pertuzumab to trastuzumab and chemotherapy [11]. A study estimating direct medical costs per patient and the cost-effectiveness of adding pertuzumab in neoadjuvant treatment for HER2-positive breast cancer indicated an increase in overall costs [34]. Pertuzumab costs alone represented a substantial portion of overall costs per patient and overall neoadjuvant therapy costs [34]. Integrating financial considerations, countries such as Canada and Italy have not funded pertuzumab in the curative setting, leading to patient disparities in treatment access and raising questions about universal access to optimal breast cancer care [11, 35]. Doležel et al. emphasized the economic challenge by highlighting the lack of cost-effectiveness in utilizing pertuzumab in breast cancer treatment [36]. Consequently, QL1209 emerges as an effective biosimilar, offering a cost-effective alternative that may alleviate financial burdens and significantly contribute to the accessibility of pertuzumab for HER2-positive breast cancer patients.

However, certain limitations should be noted. Firstly, as a major protocol version change, patients enrolled according to the latter version received adjuvant treatment with FEC chemotherapy and subsequent dual anti-HER2 treatment, enabling long-term efficacy, safety, and immunogenicity observations for QL1209. Secondly, the study concluded after surgery or adjuvant treatment, scheduled 28 days after the final administration of the study drug. Thus, the long-term follow-up data was limited, which impacts little on the scientific robustness of the primary endpoint and the equivalence of QL1209 to reference pertuzumab.

## Conclusions

This phase III study establishes the equivalence of QL1209 to reference pertuzumab in terms of both efficacy and safety. Furthermore, QL1209 exhibited comparable PK and immunogenicity to the reference pertuzumab. These findings position QL1209 as a promising new option for patients diagnosed with HER2-positive breast cancer.

### Supplementary information

Supplemental Material

## Data Availability

Data were generated by the authors and are available on request.
