# Supplementary material for: QL1209 (pertuzumab biosimilar) versus reference pertuzumab plus trastuzumab and docetaxel in neoadjuvant treatment for HER2-positive, ER/PR-negative, early or locally advanced breast cancer: A multicenter, randomized, double-blinded, parallel-controlled, phase III equivalence trial
Source: Br J Cancer. 2024 Jun 21;131(4):668–75. doi: 10.1038/s41416-024-02751-2 (PMC11333611; doi:10.1038/s41416-024-02751-2)
Supplement: Supplementary file 1 — Supplemental Material [file 41416_2024_2751_MOESM1_ESM.docx]

**Supplementary Material**

# Supplementary methods

## 1. Inclusion and exclusion criteria

### 1.1 Inclusion criteria

Subjects participated in the study only if all the following criteria were met:

1. Be willing and able to provide written informed consent for the trial;
2. Age 18-75 years;
3. Breast cancer met the following criteria: patients had histologically confirmed primary invasive adenocarcinoma of the breast, with tumor size >2 cm; with the early-stage (TNM stage T2-3, N0-1, M0) or locally advanced-stage (TNM stage N2/N3, M0, T4, and overall N and M0) according to 8th edition of the American Joint Committee on Cancer (AJCC) staging system; with HER2 positivity confirmed by molecular pathology (IHC and FISH testing); were negative for both estrogen receptor (ER) and progesterone receptor (PR) status;
4. Eastern Cooperative Oncology Group-performance status (ECOG-PS) of 0-1;
5. Baseline left ventricular ejection fraction (LVEF) ≥55% measured by echocardiography (first choice) or multigated acquisition scan;
6. All non-menopausal female (no amenorrhoea for ≥12 months) must had a negative serum pregnancy test prior to starting study treatment;
7. For non-menopausal (no amenorrhoea for ≥12 months) or non-surgically sterilised female and all male patients, should be agree to use effective contraceptive methods for themselves and their partners during the study and for 7 months after the last dose of study drug;
8. Hematology results should meet the following criteria (no blood transfusion, no use of hematopoietic factors, and no drug correction within 14 days): white blood cell count ≥3.0× 10^9^/L; absolute neutrophil count ≥1.5 × 10^9^/L; platelet count ≥100 × 10^9^/L; hemoglobin level ≥90 g/L;
9. Liver and kidney functions fulfiled the following criteria: total bilirubin ≤ 1.5 × ULN, ≤2 × ULN for subjects known to have Gilbert's syndrome; aspartate transaminase (AST) ≤2.5 × ULN; alanine transaminase (ALT) ≤2.5 × ULN; alkaline phosphatase ≤2.5 × ULN; serum creatinine ≤1.5 × ULN;
10. Coagulation function must meet: international normalized ratio (INR) ≤1.5; activated partial thromboplastin time (APTT) ≤1.5 × ULN;
11. Subjects judged by the investigator to be able to adhere to the study protocol.

### 1.2 Exclusion criteria

Subjects were ineligible for enrollment into the study if any of the following criteria were met:

1. Subjects with metastatic disease (stage IV) breast cancer;
2. Subjects with inflammatory breast cancer;
3. Subjects with bilateral breast cancer;
4. Previous history of radiation therapy, antineoplastic treatment for any malignancy;
5. Past or current history of malignant neoplasms within 5 years prior to signing the informed consent form, except for curatively treated carcinoma in situ of uterine cervix, basal cell carcinoma of the skin or squamous cell carcinoma of the skin;
6. Receiving other antineoplastic treatment, including chemotherapy, immunotherapy or Chinese proprietary medicines with clear anti-tumour indications, etc.;
7. Major surgery within 4 weeks prior to randomization and has not fully recovered from this operation;
8. Administration of any clinical study drug within 4 weeks prior to randomization;
9. Subjects who were positive for human immunodeficiency virus (HIV) and anti-hepatitis C virus (HCV) antibodies with positive HCV RNA, and test positive for syphilis antibodies before randomization, and are positive for hepatitis B surface antigen (HBsAg) with quantitative hepatitis B virus (HBV) DNA determined by the investigator according to local standards;
10. Serious cardiac illness or discomfort before randomization, including but not limited to the following diseases: history of chronic heart failure within 6 months prior to signing the informed consent form (New York Heart Association [NYHA] class II-IV); serious arrhythmias requiring treatment (except atrial fibrillation, paroxysmal supraventricular tachycardia); unstable angina; clinically significant valvular heart disease; evidence of transmural infarction on electrocardiogram (ECG); uncontrolled hypertension (systolic >160 mmHg and/or diastolic >100 mmHg);
11. Allergic to any investigational drug or any of its components or excipients, or allergic to benzyl alcohol;
12. Lactating women;
13. Subjects determined by the investigator to have complications that could interfere with the study protocol or other conditions unsuitable for participation in the clinical trial.

## 2. Statistical analysis

Analyses of the primary endpoint (tpCR) and secondary endpoints (bpCR, ORR, EFS, and DFS) were performed in the full analysis set (FAS), which was defined as all patients who were enrolled and received at least one dose of the study drug based on an intention-to-treat (ITT) basis. Safety was assessed in the safety set (SS), which included all patients who received at least one dose of the study drug and had post-dosing safety data recorded. The PK analysis was evaluated in a PK analysis set (PKAS), and covered subjects who received at least one dose of the study drug, had evaluable PK data and had no trial protocols that seriously compromised the PK evaluation. Immunogenicity analysis was in the immunogenicity set, which referred to all randomly assigned patients who received at least one dose of the study drug and had at least one post-dosing immunogenicity evaluation.

Categorical variables were presented as numbers and frequencies, and continuous variables were presented as mean and standard deviation values. Rates of tpCR, bpCR, and ORR and their 90% CIs were calculated using the Wald Confidence Limits method. For the primary endpoint, the analysis provided the frequency distribution and exact the 90% CI in each treatment group (for all patients and broken down for stratification factor), the difference in the tpCR rate between the 2 treatment groups and exact 90% CI, and the ratio of the tpCR rate in the 2 treatment groups and the 90% CI based on normal approximation. The secondary efficacy variables (bpCR and ORR) were also analyzed according to the methodology of the primary variables and adjustments were also made. Time-to-event endpoints (EFS and DFS) were estimated with the Kaplan-Meier method along with their 95% CIs and the inter-group comparisons were performed using the stratified log-rank test. Hazard ratios and associated 90% CIs were assessed with the use of a stratified Cox proportional-hazards model.

Based on the pharmacokinetic analysis set, a descriptive summary of serum trough concentrations (C_trough_) for various planned visits in different treatment groups, as well as geometric mean and geometric coefficient of variation (CV%), are provided. Generalized linear models were employed to calculate the geometric mean ratio and its 90% confidence interval (CI) for C_trough_ between treatment groups at different visits during the neoadjuvant and FEC therapy period. Additionally, the point estimate and its 90% CI for the geometric least squares mean ratio were calculated. Immunogenicity analysis was based on immunogenicity set, and summarized the number and percentage of patients who were positive (in the total population and in those with at least one positive occurrence) for anti-drug antibodies (ADA) and neutralizing antibodies (NAb). Moreover, box plots of the C_trough_ of subjects by different subgroups (ADA-negative vs. positive) were produced.

Sensitivity analyses including tipping-point and supplementary analyses (based on a supplementary analysis set of participants with positive HER2 detection results and negative ER/PR results as evaluated by the central laboratory) were conducted. Tipping-point analyses quantified the impact of unmeasured confounders and performed quantitative bias analyses for missing data.

# Supplementary results

### Table S1 PPS analysis, and supplementary analysis results of tpCR in patients with HER2-positive, ER/PR-negative determined by the central laboratory

| tpCR | QL1209 | Reference pertuzumab | Relative risk |
| --- | --- | --- | --- |
| PPS analysis | 107/232  (46.12; 40.74 to 51.50) | 115/240  (47.92; 42.61 to 53.22) | 0.96  (0.82 to 1.13) |
| Supplementary analysis | 94/225  (41.78; 36.37 to 47.19) | 105/237  (44.30; 39.00 to 49.61) | 0.94  (0.79 to 1.12) |

PPS analysis (including all patients in the FAS but except those who had a major protocol deviation or did not receive four doses of study drug at least) was performed to further validate equivalence between the two groups, and the results demonstrated that the two groups were also equivalent in the pps population. The inclusion of patients in this study was based on locally assessed HER2 status, and to reaffirm this result, we planned additional analyses of the primary efficacy endpoint based on the FAS of patients with centrally laboratory assessed HER2-positive and ER/PR-negative status. The results demonstrated that QL1209 and pertuzumab were similar. tpCR, total pathological complete response. Data are no. of patients (n, %; 90% CI).

### Table S2 Summary of C_trough_

|  | QL1209 | Reference pertuzumab |
| --- | --- | --- |
| Before the cycle 2 dosing | 68251.05 (24.02) | 59723.75 (26.21) |
| Before the cycle 3 dosing | 65070.88 (30.74) | 57672.54 (31.82) |
| Before the cycle 4 dosing | 66018.73 (31.98) | 56975.69 (33.49) |
| Before the cycle 5 dosing | 18169.72 (67.55) | 16801.41 (59.76) |

C_trough_ remained stable and similar between the two groups throughout cycles 2-4. Data are mean, ng/mL (CV, %), unless otherwise indicated; CV, coefficient of variation.

### Table S3 Summary of immunogenicity results

|  | QL1209 (n=256) | Reference pertuzumab (n=257) |
| --- | --- | --- |
| Neoadjuvant and FEC periods |  |  |
| At least one ADA positivity | 6 (2.34) | 9 (3.50) |
| At least one NAb positivity | 2 (0.78) | 3 (1.17) |
| Treatment-induced ADA positivity | 3 (1.17) | 6 (2.33) |
| At least one post-baseline NAb positivity | 2 (0.78) | 3 (1.17) |
| Entire treatment periods |  |  |
| At least one ADA positivity | 8 (3.12) | 17 (6.61) |
| At least one NAb positivity | 4 (1.56) | 11 (4.28) |
| Treatment-induced ADA positivity | 5 (1.95) | 14 (5.45) |
| At least one post-baseline NAb positivity | 4 (1.56) | 11 (4.28) |

These results indicate that QL1209 had a similar immunogenicity profile to reference pertuzumab in this study. Data are n (%), unless otherwise indicated; FEC, fluorouracil, epirubicin, and cyclophosphamide; ADA, anti-drug antibodies; NAb, neutralizing antibodies.

### Table S4 Effect of immunogenicity on pharmacokinetics

| C_trough_ (ng/mL) | QL1209 ^1^ | | Reference pertuzumab ^1^ |  |
| --- | --- | --- | --- | --- |
| Inclusion of ADA-positive patients | | | | |
| Cycle 1 | 5.08 | 5.05 | | |
| Cycle 2 | 11.08 | 10.94 | | |
| Cycle 3 | 11.01 | 10.87 | | |
| Cycle 4 | 11.02 | 10.85 | | |
| Exclusion of ADA-positive patients | | | | |
| Cycle 1 | 5.08 | 5.05 | | |
| Cycle 2 | 11.09 | 10.97 | | |
| Cycle 3 | 11.03 | 10.92 | | |
| Cycle 4 | 11.04 | 10.91 | | |
| Inclusion of Nab-positive patients | | | | |
| Cycle 1 | 5.08 | 5.05 | | |
| Cycle 2 | 11.08 | 10.94 | | |
| Cycle 3 | 11.01 | 10.87 | | |
| Cycle 4 | 11.02 | 10.85 | | |
| Exclusion of Nab-positive patients | | | | |
| Cycle 1 | 5.08 | 5.05 | | |
| Cycle 2 | 11.09 | 10.96 | | |
| Cycle 3 | 11.03 | 10.92 | | |
| Cycle 4 | 9.40 | 9.54 | | |

After taking the ADA and NAb into control (inclusion or exclusion patients with ADA/Nab positive), the pharmacokinetic parameters were still similar, suggesting that ADA do not affect the PK values. 1, Value means geometric least squares mean; ADA, anti-drug antibodies; Nab, neutralizing antibodies.

### Table S5 Effect of immunogenicity on efficacy

| tpCR | QL1209 group,  n (%) | Reference pertuzumab, n (%) |
| --- | --- | --- |
| Exclusion of ADA-positive patients | 105/246 (42.68) | 112/242 (46.67) |
| Exclusion of NAb-positive patients | 107/250 (42.80) | 115/246 (46.75) |

After taking the ADA and NAb into control (exclusion patients with ADA/Nab positive), the tpCR was still similar, suggesting that ADA do not affect the efficacy. ADA, anti-drug antibodies; Nab, neutralizing antibodies; tpCR, total pathological complete response.

### Table S6 Effect of immunogenicity on adverse events

|  | QL1209 group, n (%) | Reference pertuzumab, n (%) |
| --- | --- | --- |
| ADA-positive | n = 8 | n = 17 |
| Any TEAEs | 8 (100) | 16 (94.12) |
| TRAEs | 8 (100) | 16 (94.12) |
| Grade ≥3 TRAEs | 4 (50) | 3 (17.65) |
| Serious TRAEs | 1 (12.50) | 0 |
| ADA-negative | n = 248 | n = 240 |
| Any TEAEs | 236 (95.16) | 232 (96.67) |
| TRAEs | 194 (78.23) | 191 (79.58) |
| Grade ≥3 TRAEs | 56 (22.58) | 54 (22.50) |
| Serious TRAEs | 9 (3.63) | 10 (4.17) |
| NAb-positive | n = 4 | n = 11 |
| Any TEAEs | 4 (100) | 11 (100) |
| TRAEs | 4 (100) | 11 (100) |
| Grade ≥3 TRAEs | 2 (50) | 3(27.27) |
| Serious TRAEs | 1 (25) | 0 |
| NAb-negative | n = 252 | n = 246 |
| Any TEAEs | 240 (95.24) | 237 (96.34) |
| TRAEs | 198 (78.57) | 196 (79.67) |
| Grade ≥3 TRAEs | 58 (23.02) | 54 (21.95) |
| Serious TRAEs | 9 (3.57) | 10 (4.07) |

The adverse events were still similar in ADA-positive, ADA-negative, NAb-positive, and NAb-negative population, suggesting that ADA do not affect the adverse events. ADA, anti-drug antibodies; NAb, neutralizing antibodies; tpCR, total pathological complete response. TEAE, treatment-emergent adverse event; TRAE, treatment-related adverse event; ADA, anti-drug antibodies.

### Figure S1 Tipping ponit analysis of tpCR as assessed by IRC


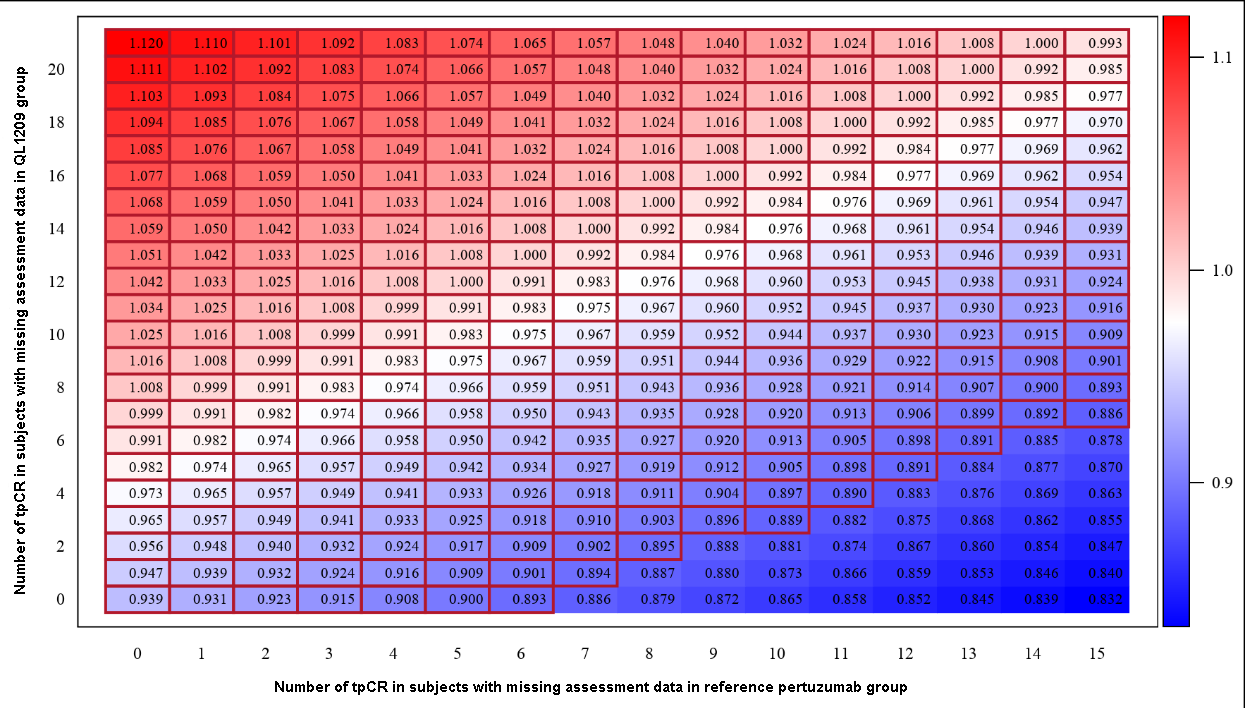


A post-hoc tipping point analysis on missing data was conducted. The conclusion regarding the robustness of the equivalence study is upheld only in extreme scenarios, such as when at least 7 out of 15 subjects with missing primary efficacy indicators in the control group respond, and all 21 subjects with missing primary efficacy indicators in the experimental group show no response (denoted as: R≥7/15, T0/21). Alternatively, the reversal of the conclusion occurs only in situations where extreme conditions are met, such as R≥8/15, T≤1/21; R≥9/15, T≤2/21; R≥10/15, T≤2/21; R≥11/15, T≤3/21; R≥12/15, T≤4/21; R≥13/15, T≤5/21; R≥14/15, T≤6/21; or R≥15/15, T≤6/21. This provides evidence of the robustness of the conclusion regarding the equivalence of the study. IRC, independent review committee.

### Figure S2 Event-free survival and disease-free survival plots (full analysis set)


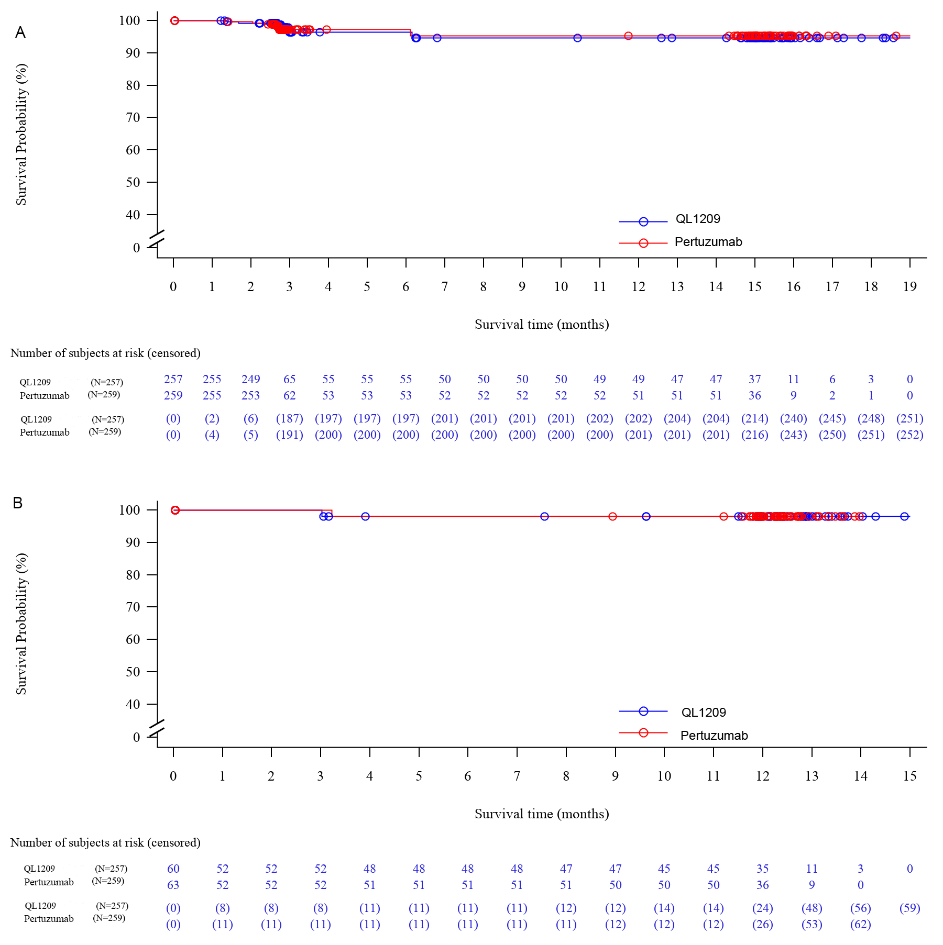


A, event-free survival in the two groups; B, disease-free survival in the two groups.

### Figure S3 Mean C_trough_-time plot between two groups (pharmacokinetic analysis set)


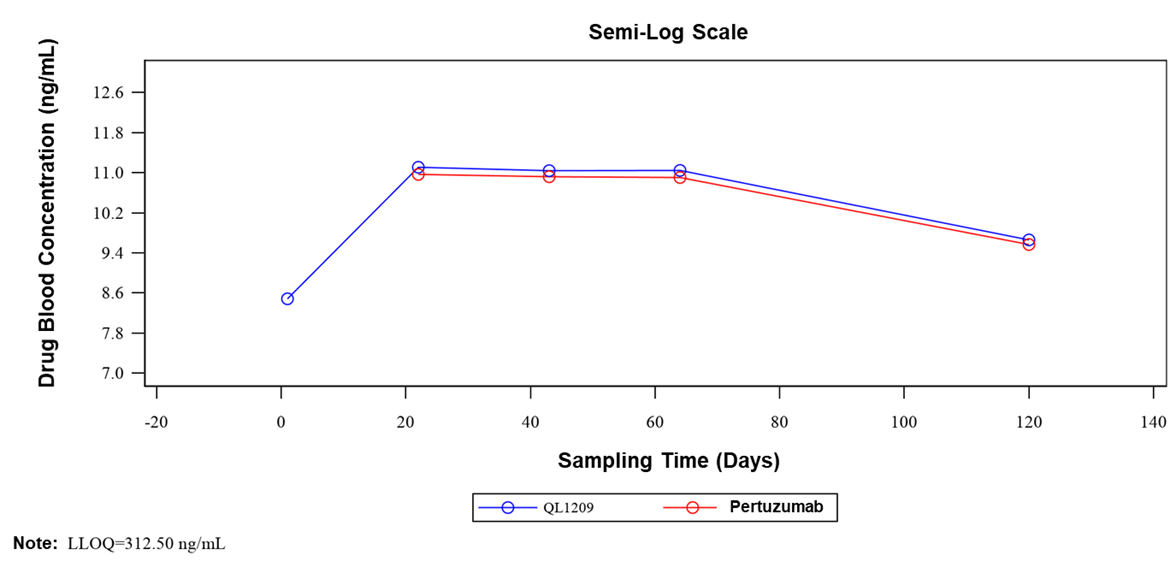


From cycles 1 to 4 during the neoadjuvant treatment, the mean drug blood concentration (trough concentration) was similar between QL1209 and pertuzumab groups. The pharmacokinetic profile of QL1209 was similar to that of pertuzumab.
